# Calcium Oxalate Crystals in Leaves of the Extremophile Plant *Colobanthus quitensis* (Kunth) Bartl. (Caryophyllaceae)

**DOI:** 10.3390/plants10091787

**Published:** 2021-08-27

**Authors:** Olman Gómez-Espinoza, Daniel González-Ramírez, Jairo Méndez-Gómez, Rossy Guillén-Watson, Alejandro Medaglia-Mata, León A. Bravo

**Affiliations:** 1 Laboratorio de Fisiología y Biología Molecular Vegetal, Instituto de Agroindustria, Departamento de Ciencias Agronómicas y Recursos Naturales, Facultad de Ciencias Agropecuarias y Forestales & Center of Plant, Soil Interaction and Natural Resources Biotechnology, Scientific and Technological Bioresource Nucleus, Universidad de La Frontera, Temuco 1145, Chile; o.gomez01@ufromail.cl or; 2 Centro de Investigación en Biotecnología, Escuela de Biología, Instituto Tecnológico de Costa Rica, Cartago 30101, Costa Rica; daniel.agr13@estudiantec.cr (D.G.-R.); roguillen@itcr.ac.cr (R.G.-W.); 3 Laboratorio Institucional de Microscopía, Instituto Tecnológico de Costa Rica, Cartago 30101, Costa Rica; jamego8.4.97@gmail.com (J.M.-G.); amedaglia@itcr.ac.cr (A.M.-M.)

**Keywords:** Antarctic, ecotypes, crystal idioblasts, druses, Caryophyllaceae

## Abstract

The presence of calcium oxalate (CaOx) crystals has been widely reported in the plant kingdom. These structures play a central role in various physiological functions, including calcium regulation, metal detoxification, and photosynthesis. However, precise knowledge about their possible roles and functions in plants is still limited. Therefore, the present work aims to study the ecotypic variability of *Colobanthus quitensis*, an extremophile species, concerning CaOx crystal accumulation. The CaOx crystals were studied in leaves of *C. quitensis* collected from different provenances within a latitudinal gradient (From Andes mountains in central Chile to Antarctica) and grown under common garden conditions. Polarized light microscopy, digital image analysis, and electron microscopy were used to characterize CaOx crystals. The presence of CaOx crystals was confirmed in the four provenances of *C. quitensis*, with significant differences in the accumulation among them. The Andean populations presented the highest accumulation of crystals and the Antarctic population the lowest. Electron microscopy showed that CaOx crystals in *C. quitensis* are classified as druses based on their morphology. The differences found could be linked to processes of ecotypic differentiation and plant adaptation to harsh environments.

## 1. Introduction

Calcium oxalate (CaOx) is an insoluble salt formed when oxalic acid ([HOOC.COOH]) combines with calcium ions (Ca^2+^) and then precipitates. Calcium oxalate mineralization is typical in land plants, fungi, lichens, animals, and sparingly in three divisions of algae [1]. Calcium oxalate is an anti-nutritional agent in animals and humans that stimulates certain diseases such as hyperoxaluria or urolithiasis. Opposing, in photosynthetic organisms, the accumulation of calcium oxalate crystals at high levels is a common activity tightly integrated with metabolism [1,2,3]. CaOx crystals are widespread throughout the plant kingdom; they have been detected in at least 215 families and represent in many cases more than 90% of the calcium in plant bodies [4,5]. 

CaOx mineralization in plants is considered to be a highly regulated process by which intracellular crystals of defined morphologies are deposited in the vacuoles of specialized cells known as crystal idioblasts [1]. CaOx crystals are present in all plant organs; in leaves, crystals idioblasts are mainly located within mesophyll and bundle sheath extensions [6,7]. Crystal morphology is diverse, including individual needle-like styloid, large single prisms, acicular raphides occurring in bundles, crystal sands consisting of many tiny individual prisms, and druses, which are aggregates of numerous prismatic elements [8].

Anton van Leeuwenhoek (1675) initially described the CaOx crystals in his early microscopic observations of plant cells. Many functions have since been attributed to them, some of which have been very speculative [4,9]. Among the possible roles of CaOx crystals, calcium regulation and homeostasis and calcium reserve are the most complete studied. Other promising functions include heavy metal detoxification and a source of CO_2_ for photosynthesis (alarm photosynthesis, AP) [2,9]. Furthermore, recent evidence showed that CaOx crystals represent multifunctional tools essential for plants, especially under stress conditions [10]. A detailed description of each possible CaOx function can be found in the reviews made by Franceschi and Nakata (2005) [3] and Paiva (2021), (2019) [4,9]. 

Among the assigned functions of CaOx crystals, the recent findings on AP, the use of CaOx as a rich source of CO_2_ for photosynthesis, could provide valued information for developing innovative tools regarding plant stress tolerance [10]. In this context, Gómez-Espinoza et al. (2020) studied the relationship between CO_2_ limiting conditions and CaOx crystal decomposition in the Antarctic plant *Colobanthus quitensis* [11]. They concluded that CaOx crystals in this species display diurnal fluctuations and are associated as an internal CO_2_ source for carbon requirements for photosynthesis [11]. However, the occurrence and morphology of CaOx crystals in the leaves of *C. quitensis* have not been appropriately reported thus far.

*Colobanthus quitensis* (Kunth) Bartl. (Caryophyllaceae) is a eudicotyledon, extremophile, stress-tolerant plant that inhabits low temperature environments from southern Mexico to maritime Antarctica. Given its wide geographical distribution and the isolation in which various populations of this species have developed, ecotypes adapted to particular environments have been generated [12,13,14,15]. The *C. quitensis* populations or geo-ecotypes show morphological and genetic variability, which is attributed to continuous selection processes in response to the environmental conditions prevailing in each habitat [16]. Previous studies using *C. quitensis* populations from a latitudinal gradient showed that the ecotypes maintain their geographical characteristics even in controlled cultivation conditions in a common garden [16]. Furthermore, the high-latitude populations and low-latitude populations generally presented the most contrasting anatomical traits [17]. Therefore, these trait variations within the populations correspond to ecotypic genetic adaptation rather than phenotypic plasticity.

The most studied ecotypes are found in the maritime Antarctic, the maritime regions of Punta Arenas, and the Andean Mountains in Chile [16,18,19]. This represents a gradient of 29° of latitude and a linear distance of about 3350 km between the extremes. Thermal gradients associated with this latitudinal gradient have been shown elsewhere [16]. The presence of CaOx crystals has been reported in the *C. quitensis* Antarctic population [20]. However, to our knowledge, it is unknown if the other ecotypes also share these structures in their leaves or whether there is an association of the crystal presence with a gradient of harsher environmental conditions. Therefore, in the present study, the types, morphology, and location of calcium oxalate crystals in the leaves of four *C. quitensis* populations (representing a latitudinal gradient from central Chile to Antarctica) were investigated in a common garden experiment. According to the literature, *C. quitensis* plants grown at low temperatures [21] or populations developed at higher latitudes [22] exhibit constitutively more dense leaves with higher CO_2_ diffusion limitations. If this is the case, we would expect that populations developed at higher latitudes (lower average growth temperature and general harsher environmental conditions) would exhibit smaller and less abundant CaOx crystals than lower latitude populations in the middle of the photoperiod. 

## 2. Results and Discussion

### 2.1. Differences in Colobanthus quitensis Provenances

**Figure 1:**
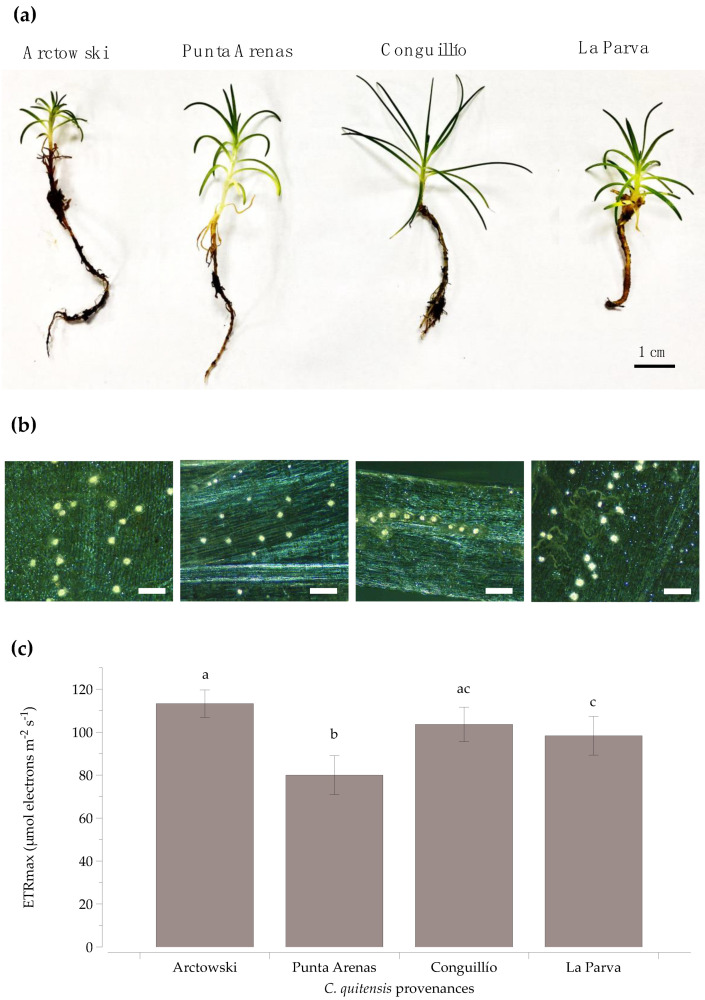
Anatomical and photosynthetic traits of four *Colobanthus quitensis* provenances (**a**) A habit from the four provenances of *C. quitensis*, from left to right showing a south-north pattern.; (**b**) Paradermal view of the chlorine-bleached leaves from four provenances of *C. quitensis* under polarized light (10X). CaOx crystals are visible as bright spots. The micrographs follow the same provenances pattern as in (**a**). The white scale bar: 200 µm.; (**c**) Maximum electron transport capacity (ETRmax) at light saturation from the four provenances of *C. quitensis.* Plants were grown under common garden conditions. Error bars denote SE of mean; n = 10. Different letters represent statistically significant differences between provenances (one-way ANOVA; *p* < 0.05).

The studied *C. quitensis* provenances exhibited visible morphological variations such as habit size and differences in leaf length-width, even when plants were grown in the same controlled cultivation conditions in a common garden (Figure 1a and Figure S1). For example, plants from the Arctowski population had the smallest shoots and the smallest leaf length among ecotypes. In contrast, the Conguillío plants presented the largest shoots and leaves but also the narrowest leaves. Thus, the morphological characteristics showed by the ecotypes agree with those obtained by Cuba-Díaz et al. (2017), where the Conguillío and Arctowski (Antarctic) populations presented the most contrasting anatomical traits, and the Punta Arenas and La Parva ecotypes showed intermediate-sized structural features [17]. 

Polarized light photomicrographs revealed CaOx crystal structures in the leaves of the four provenances of *C. quitensis*. The CaOx crystals were clearly visible as bright spots within the leaf due to their optical property of birefringence (Figure 1b). Crystals were distributed throughout the entire leaf in plants from four studied provenances, without a defined pattern but closer to the central vein. The obtained results represent a novelty since the occurrence of these crystals had only been reported in leaves from the Antarctic ecotype [20,23].

Chlorophyll fluorescence (ChlF) analysis was performed to investigate whether the observed anatomical differences in the *C. quitensis* provenances still maintain at the physiological level. Contrary to their small anatomical features, plants from the Arctowski population showed the highest values of maximum electron transport capacity (ETRmax), being statistically higher than those displayed by Punta Arenas and La Parva ecotypes. The Conguillío plants also showed high ETRmax values, but without significant differences from the Arctowski and La Parva plants. In contrast, plants from Punta Arenas showed statistically lower ETRmax values compared to all ecotypes. 

Previous reports of ChlF measurements on *C. quitensis* populations were exclusively focused in comparison with the Arctowski and La Parva populations under specific situations such as high UV-B radiation, low temperature, cold acclimation, and light intensity [14,24,25,26]. In general, in those studies, as with ours, Antarctic individuals showed higher photosynthetic performance than plants from La Parva. Nevertheless, to our knowledge there are no reports of ChlF or photosynthesis (*A_N_*) in the Punta Arenas and Conguillío populations. Further experiments are needed to understand the adaptations that these populations have adopted that explain these electron transport rate differences; they may involve photosynthetic capacity or different alternative electron sinks. 

Tukey’s post hoc test showed significant differences between the ETR values of plants from the Andean Mountains (Conguillío and La Parva) and those from Antarctica, at all photosynthetically active radiation (PAR) intensities applied in the light response curves (LRCs) (Figure S2). The differences observed in the LRCs and the ETRmax of each procedence are consistent with the ecotypic differentiation in *C. quitensis* as reported by Gianoli et al. (2004) [15,27]. In this sense, the *C. quitensis* from the La Parva and Antarctic ecotypes have contrasting adaptations to climatic conditions. In the Andean plants, its strategy aims to reduce the probability of capturing light by having smaller chloroplasts and a higher level of thermal dissipation. In contrast, the Antarctic ecotype has a smaller antenna/core ratio of PSII, higher photochemical utilization of the absorbed energy with lower sensitivity to low temperatures, and higher levels of photoprotective pigments [28].

### 2.2. CaOx Crystal Presence in C. quitensis Leaves

**Figure 2:**
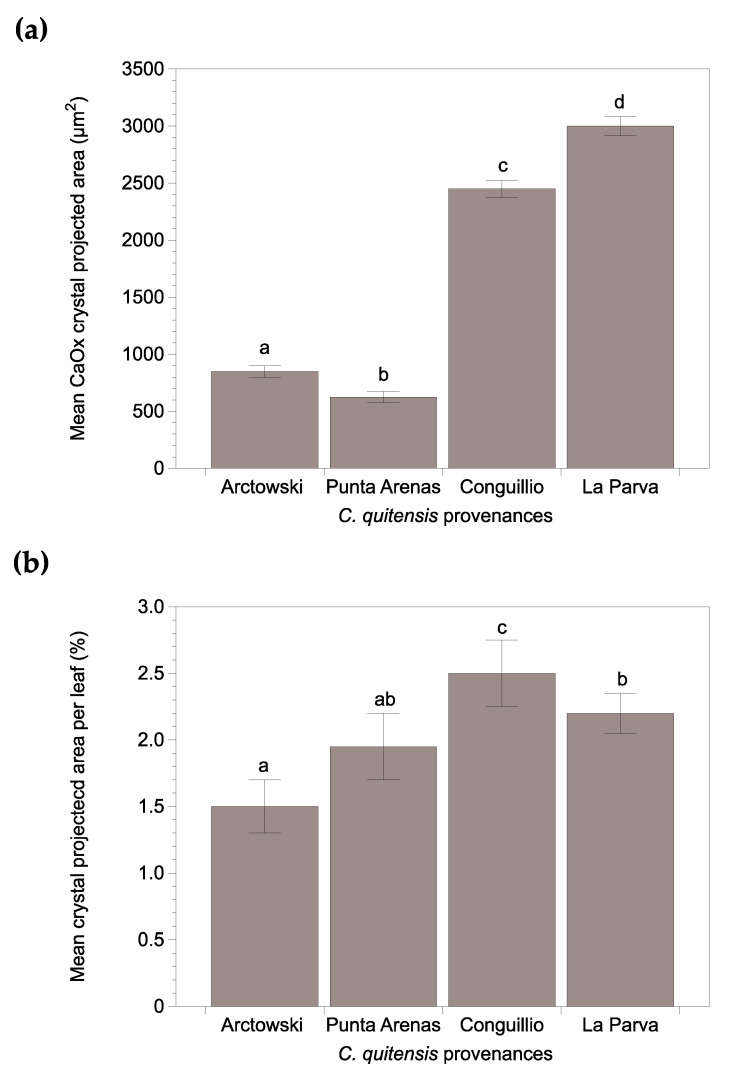
Calcium oxalate crystals accumulation in four provenances of *Colobanthus quitensis* (**a**) Mean CaOx crystals projected area and; (**b**) Relative projected area of CaOx crystals in the leaves of four provenances of *C. quitensis*. Error bars denote SE of mean; n = 5 cushions (10 leaves per cushion). Different letters represent statistically significant differences between provenances (One-way ANOVA; *p* < 0.05).

Significant differences in CaOx crystal size were evidenced among *C. quitensis* provenances (Figure 2a). The dimensions of the crystals varied between 500 µm^2^ in the Arctowski plants and 3000 µm^2^ in plants from La Parva. Furthermore, significant differences in crystal accumulation between ecotypes were also observed (Figure 2b). The bigger CaOx crystals and the most crystal area per leaf were evidenced in both Andean plants. The Conguillio plants showed the highest mean values, while the Arctowski plants presented the lowest mean. La Parva and Punta Arenas plants showed intermediate values. These obtained results agreed with the south-north gradient pattern studied by Cuba et al. (2017) [17], where the southernmost populations have smaller morphological traits. 

Until now, the genes that control CaOx crystals’ biosynthetic and degradation pathways have not been well-documented in plants [29]. Therefore, there is no gene expression evidence for differences in the size and accumulation of crystals in the leaves of *C. quitensis* populations. The evidence that plants coming from populations that are more than 3500 km away, are grown in a common garden, are well irrigated and fertilized, still exhibited differential accumulation of CaOx crystals. This strongly suggests that this trait is not related to the phenotypic plasticity of this species. Therefore, as it has been reported for other anatomical traits, these variations may be related to the genetic differentiation found among *C. quitensis* ecotypes. They may be attributed to continuous selection processes, where several characteristics may vary in response to environmental conditions prevailing in each specific habitat [16]. 

Within the studied provenances, significant differences in the environmental characteristics of each habitat are noteworthy. For example, La Parva and Conguillío (with the larger crystals) both inhabit mountainous areas (3600 and 2500 m a.s.l., respectively), the former in a mild Mediterranean climate with precipitation between 400 and 900 mm annually (3 to 10 °C); while Conguillío inhabits sites with high volcanic activity and have a contrasting annual rainfall of 2500–3000 mm (−1 to 15 °C). In contrast, Punta Arenas and Arctowski both inhabit maritime zones, with a maximum altitude of 3 m a.s.l. for Punta Arenas and 23 m a.s.l. for the Antarctic population. The Punta Arenas ecotype is associated with humid environments, both freshwater and brackish, and soils with clay (−10 to 25 °C). Plants at Arctowski inhabit sandy soils and are exposed to strong winds (−10 to 15 °C) [16]. 

Point mutations at different loci could be another hypothesis that may explain the observed differences in CaOx crystal accumulation between populations. It has been reported that several loci can affect calcium oxalate accumulation in mesophyll cells [30]. In *C. quitensis*, previous studies have detected genetic polymorphisms between individuals from the same population and genetic variations between ecotypes, especially a significant differentiation between populations from northern and southern Chile and plants from Conguillío [19]. Therefore, ecotypes of *C. quitensis*, being adapted to different regions throughout continental Chile and Antarctica, could have undergone genetic polymorphisms in loci responsible for crystal formation that are causing differences in both CaOx accumulation and crystal size. In this context, studies on *Medicago truncatula* plants, mutagenized with ethyl methanesulfonate, have shown that a point mutation could lead to multiple variations in the sizes and shapes of CaOx crystals [31].

The most prevalent hypothesis for CaOx crystals’ function concerns the regulation, sequestration, or excretion of cytosolic Ca ions and/or ion balance maintenance. In this sense, several reports have suggested a positive correlation between calcium levels in substrate and calcium in plant tissues. Similarly, there is a positive relationship between calcium in plant organs and transpiration [4,9]. While the current research did not evaluate or study calcium flux, nor transpiration, we cannot rule out that these factors may explain the observed differences in CaOx accumulation. However, in the experiment, this hypothesis is thought to have a low probability of explaining these observed differences between populations. This is because the plants were not subjected to nutritional stress, since they were fertilized correctly. 

Furthermore, the common garden experiment allowed studying local adaptation without the confounding effect of phenotypic plasticity [32]. Therefore, it is not expected that some populations suffered more transpiration or calcium deficiencies than others. In addition, all the plants used were approximately the same developmental stage, so the leaf age factor should not be an influence. 

### 2.3. Scanning Electron Microscopy

**Figure 3:**
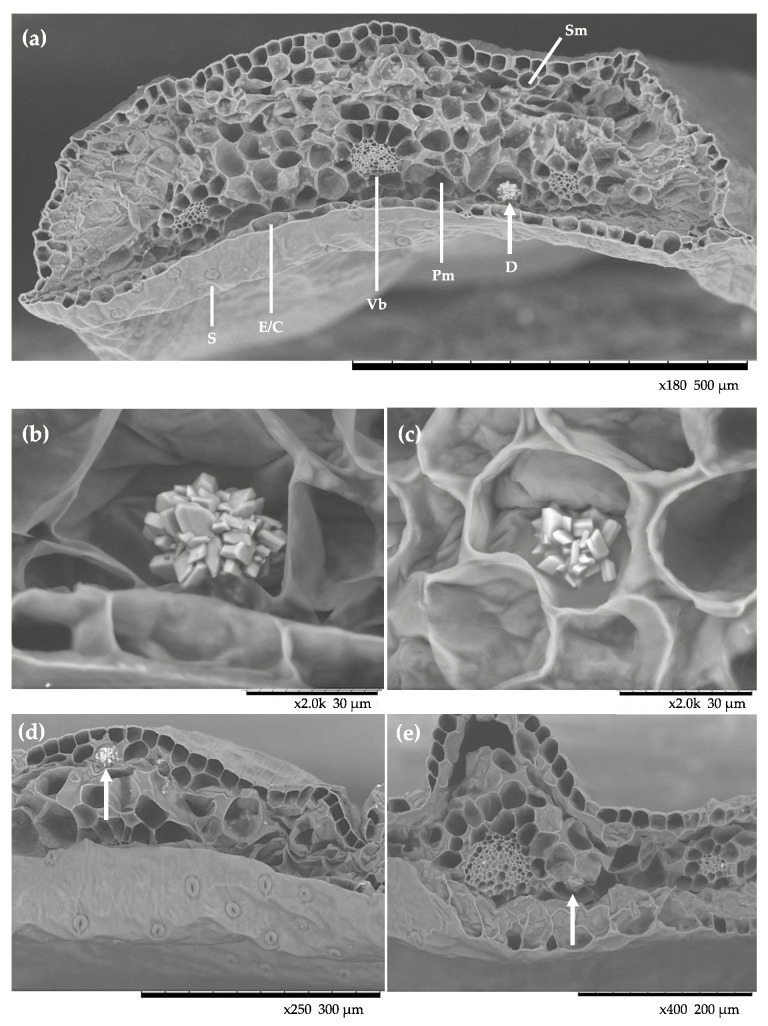
Scanning electron micrographs of calcium oxalate crystals in the leaf of *Colobanthus quitensis* Antarctic (Arctowski) ecotype. (**a**) Scanning electron micrograph showing a transverse section of *C. quitensis* leaf. A sizeable prickly crystal (white arrow) is located within mesophyll, near the vascular bundle.; (**b**) Amplification of the crystal observed in (**a**); (**c**) CaOx druse inside an idioblast.; (**d**,**e**) CaOx druses within mesophyll. In (**a**), Sm, Spongy mesophyll; S, Stoma; E, Epidermis; C, Cuticle; Vb, Vascular bundle; Pm, Palisade mesophyll; D, CaOx Druse.

**Figure 4:**
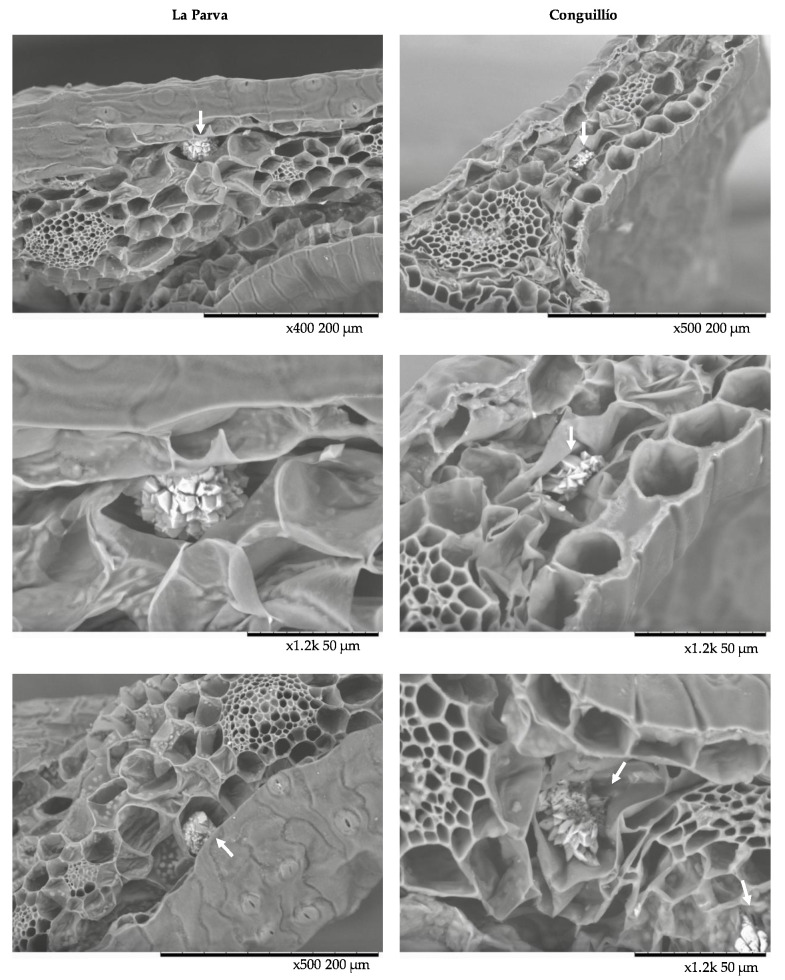
Scanning electron micrographs of calcium oxalate crystals in the leaf of *Colobanthus quitensis* from La Parva and Conguillío populations. Sizeable prickly crystals (white arrow) are located within mesophyll, near the vascular bundle.

Deeper digital micrographs of the *C. quitensis* CaOx crystals were also taken using scanning electron microscopy (SEM) (Figure 3; Figure 4). The protocol of dehydrating the samples with a gradient of acetone and drying them to a critical point allowed the obtaining of high-quality images that showed the leaf anatomy with great clarity and without collapsing the tissue or damaging its integrity (Figure S3). However, through this procedure, it was not possible to obtain images of the CaOx crystals. Therefore, we performed a variant using fresh fixed leaves without processing, immersed for seconds in liquid nitrogen, and manually cryo-fragmented. This procedure increased the possibility of finding CaOx crystals in the samples. Although some parts of the tissue sometimes collapsed, the crystals were found to be intact. Therefore, we consider that for the study of CaOx crystals in *C. quitensis* this methodology should be used.

The images showed that CaOx crystals in *C. quitensis* exhibited a druse-like morphology. In older literature, druse crystals are also known as “star crystals.” Druses exhibit a prickly appearance (Figure 3a,b). They consist of monoclinic crystals originating from a common nucleation center with sharp crystal corners pointing radially outward [8,33]. In three of the four studied populations, CaOx druses were found in the spongy tissue near the veins. The diameter of the druses ranged from 30 µm to 45 µm, which is within the range of sizes determined with light microscopy. The presence of druse-like calcium oxalate crystals in Caryophyllaceae is not rare; there have been reports of the existence of these structures in other plants of this family, such as *Silene thymifolia*, *Saponaria officinalis*, *Saponaria pamphylica* [34,35,36], as well as numerous Cactaceae members [8].

Unfortunately, the plant material of the Punta Arenas ecotype was damaged in processing, preventing the observation of CaOx crystals. The micrographs also permitted the observation of the leaf anatomy of *C. quitensis* (Figure 3a). The triangular-shaped section presents characteristics that match with those previously reported for this plant [20]. A detailed leaf micromorphology study of Antarctic *Colobanthus quitensis*, using SEM, was performed by Mantovani & Vieira (2000) [23]. In their work, the authors reported that oxalate crystals were found only in spongy cells, mainly near veins, which is consistent with our results. However, no digital micrographs of these structures were shown at the time. 

The functions of calcium oxalate crystals in *C. quitensis* are still not entirely known, even though this plant has a large number of these structures in its leaves. Gómez-Espinoza et al. (2020) suggest that the CaOx crystals in *C. quitensis* are a dynamic system which responds to environmental stimuli, such as the limitation of CO_2_, and that druses fluctuate in the course of a day. The authors found that *C. quitensis* plants exposed to a CO_2_ limitation significantly increased the CaOx crystal decomposition; they hypothesized that this decomposition is a complementary endogenous mechanism that could facilitate CO_2_ under stress situations [11]. CaOx decomposition is likely part of the adaptation to harsher environmental conditions, such as lower average and minimum temperature. Hence, the smaller size and number of CaOx crystals at the middle of the photoperiod in the provenances with the harsher environments are consistent with higher CaOx decomposition and the release of CO_2_ within the mesophyll from those plants. Thus, this release of CO_2_ may partially counterbalance the CO_2_ diffusional constraint for photosynthesis imposed at the mesophyll level in low-temperature-grown plants of this species [21]. 

In this context, the occurrence of CaOx crystals at the interspecific level seems to be climate-related [10]. For example, Acacia plants from four climate zones along an aridity gradient were investigated for CaOx crystal occurrence. The shapes of crystals were constant between species from different climate zones, implying that morphology was not affected by climate conditions (rainfall). However, as in *C. quitensis*, the distribution and accumulation of CaOx crystals did appear to be shaped by the prevailing environmental condition on their provenances [37]. Nevertheless, questions about the accumulation and function of CaOx crystals in the different ecotypes of *C. quitensis* remain open. Therefore, there is a motivation to study further the relations between the geographic distribution of *C. quitensis* populations and the accumulation of CaOx crystals, their functions, and the genetic basis governing their formation and decomposition. Here we demonstrate the abundance and occurrence of calcium oxalate crystals in the extremophilic plant *C. quitensis,* with significant differences in the accumulation between plant provenances. Changes in CaOx crystal accumulation were associated with the provenance of the plant population within the latitudinal gradient of this species and appear to be associated with the prevailing climatic conditions in which each population developed. The diversity of functions attributed to these structures is an incentive to focus research efforts towards the understanding of the role of these crystals in plant physiology and stress tolerance mechanisms. 

## 3. Materials and Methods

### 3.1. Plant Material and Growth Conditions

The calcium oxalate crystals were studied in leaves collected from *C. quitensis* plants from four populations: Arctowski (62°9′49.30″S; 58°28′8.97″W), Punta Arenas (53°22′3.20″S; 70°58′28.90″W), Conguillío (38°36′0.00″S; 71°35′60.00W), and La Parva (33°19′49.77″S; 70°16′36.94″W). Environmental characteristics from each population site can be found in Cuba-Díaz et al (2019) [16]. These plants were previously collected in the field, reproduced vegetatively in plastic pots (5 × 5 × 5 cm) in a common garden using a soil/peat/vermiculite mixture (3:1:2), and maintained in a greenhouse until having a regular-size cushion (~3 months). Plants were watered to full soil capacity every 2–3 days and fertilized once a week with 0.2 g L^−1^ Phostrogen. Leaves for crystal measurements were developed under common garden condition. Leaf samples are composed by fully expanded leaves, collected at 15:00 h (after 8 hours of light exposure). 

### 3.2. Chlorophyll Fluorescence

Ten cushions of each *C. quitensis* populations were used to obtain Rapid light-response curves (RLC) of electron transport rate (ETR) using a Maxi-Imaging-PAM Chlorophyll Fluorimeter (Walz, Effeltrich, Germany). The measurements were performed at 16 °C, at 11 different intensities of actinic light (AL) (from 23 to 1524 μmol quanta m^−2^ s^−1^); each intensity was applied for three minutes followed by a saturation pulse (6000 μmol photons m^−2^ s^−1^, 800 ms). Ten areas of interest (AOI) were selected from each cushion for the calculation of the chlorophyll fluorescence parameters. ETR was calculated according to Gómez-Espinoza et al. (2020) [11]. The maximum electron transport capacity (ETRmax) at light saturation was calculated using the Aquation ETR Curve Fitter Excel template [38].

### 3.3. CaOx Crystals Measurements in the Leaves

Leaf samples from the four *C. quitensis* populations were collected for the CaOx crystal projected area measurements. The collected leaves were bleached in sodium hypochlorite solution (5% p/p) in accordance with Gómez-Espinoza et al. (2020) [11]. Whole mature leaves were briefly put in an aqueous solution of commercial bleach for 48 h until full depigmentation. Depigmented leaves were rinsed with abundant distillated water and then put between two microscope slides (necessary to exert pressure and fully expand the leaves). Samples were observed under a light microscope adapted with a polarizing filter at 10× magnification using a Leica DM750-Camera and Leica ICC50W (Leica Microsystems, Wetzlar, Hesse, Germany). Several images were taken per leaf, covering the total leaf area. The area of each crystal was calculated by digital image analysis (ImageJ-Fiji v 2.0.0-rc69/1.52i) [39]. For each individual leaf, several images were taken comprising the total leaf area. Each individual image was analyzed as follows: (1) the image was converted to 8 bits, (2) the 8-bit image was converted to Mask, and (3) the tool “Analyzing Particles” was used to count and measure the area of the crystals in the picture using the following parameters: Size 400–5000 pixel ^2^, Circularity 0.35–1.00. The total counts (crystals area) of all images from an individual leaf were added together to obtain the total area of crystals per leaf, after which this value was divided by the total leaf area to obtain a ratio area of crystals/area leaf. At least five cushions per population were used to collect leaves (n). For each cushion 10 leaves were measured.

### 3.4. Electron Microscopy

For scanning electron microscopy (SEM), *C. quitensis* leaves were fixed in Karnovsky fixative until processing [40]. After that, the leaves were cut into small pieces, dehydrated with a 30 to 100% acetone gradient, critical point dried, and mounted on stubs with self-adhesive double-sided carbon discs [2]. Additionally, fixed leaves (without processing) were submerged in liquid nitrogen for five seconds, manually fractured, and mounted on stubs with self-adhesive double-sided carbon discs. Observations and digital micrographs were taken with a Hitachi TM3000-TableTop SEM at 15 kV.

### 3.5. Statistical Analysis

One-way analysis of variance (ANOVA) at a 95% level of significance (*p* < 0.05) was applied using JASP software (Version 0.14.1) to analyze one factor multiple comparation [41]. A Tukey Post Hoc Test was carried out in those cases where ANOVA was significant. The assumption of data normality and homoscedasticity were tested with the Shapiro–Wilk and Levene’s test.

## Supplementary Materials

The following are available online at https://www.mdpi.com/article/10.3390/plants10091787/s1, Figure S1: Cushions of different provenances of *Colobanthus quitensis* (a) Arctowski, (b) Punta Arenas, (c) La Parva, (d) Conguillío, Figure S2: Light response curves of 4 ecotypes of *C. quitensis* at 16 °C. Each point represents the mean value ± standard deviation (n = 10). Asterisks indicate significant differences between at least two of the means (*p* < 0.05), Figure S3: Scanning electron micrograph showing a transverse section of *C. quitensis* leaf (a). A sizeable stoma is visible in (b) and vascular bundle in (c).
