# Supplementary material for: Calcium Oxalate Crystals in Leaves of the Extremophile Plant Colobanthus quitensis (Kunth) Bartl. (Caryophyllaceae)
Source: Plants (Basel). 2021 Aug 27;10(9):1787. doi: 10.3390/plants10091787 (PMC8470922; doi:10.3390/plants10091787)
Supplement: Supplementary file 1 [file plants-10-01787-s001.zip › plants-1340787-SI.pdf]

plants
MDPI

Supplementary materials

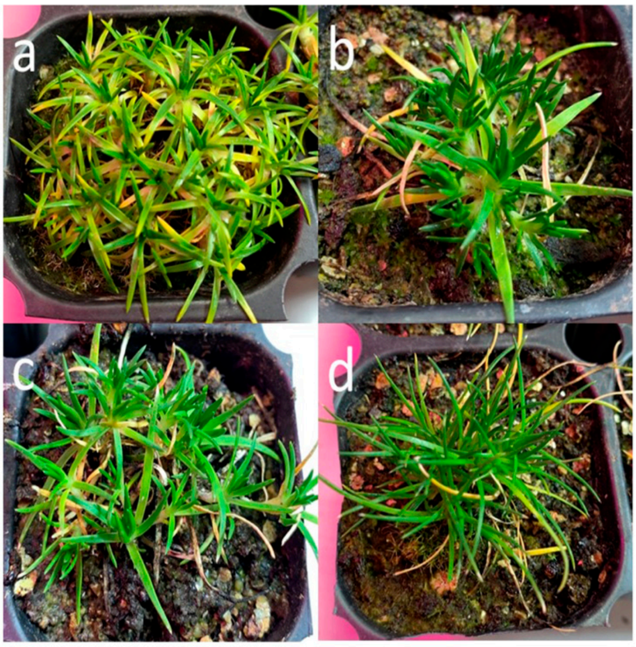

Figure S1. Cushions of different provenances of *Colobanthus quitensis* (**a**) Arctowski, (**b**) Punta Arenas, (**c**) La Parva, (**d**) Conguillío.

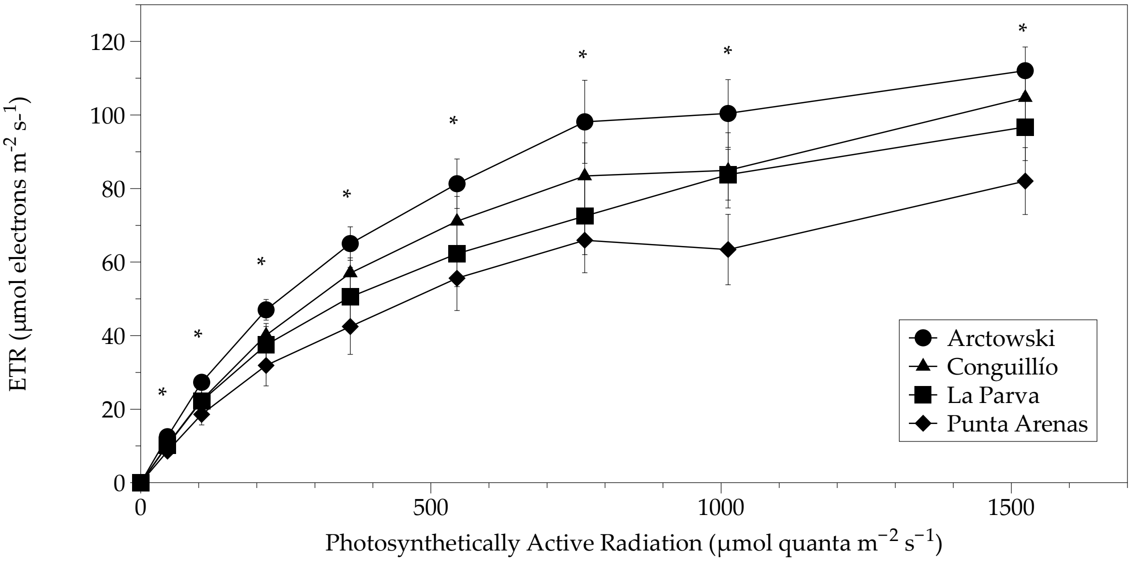

Figure S2. Light response curves of 4 ecotypes of *C. quitensis* at 16 °C. Each point represents the mean value ± standard deviation (n = 10). Asterisks indicate significant differences between at least two of the means ($p < 0.05$).

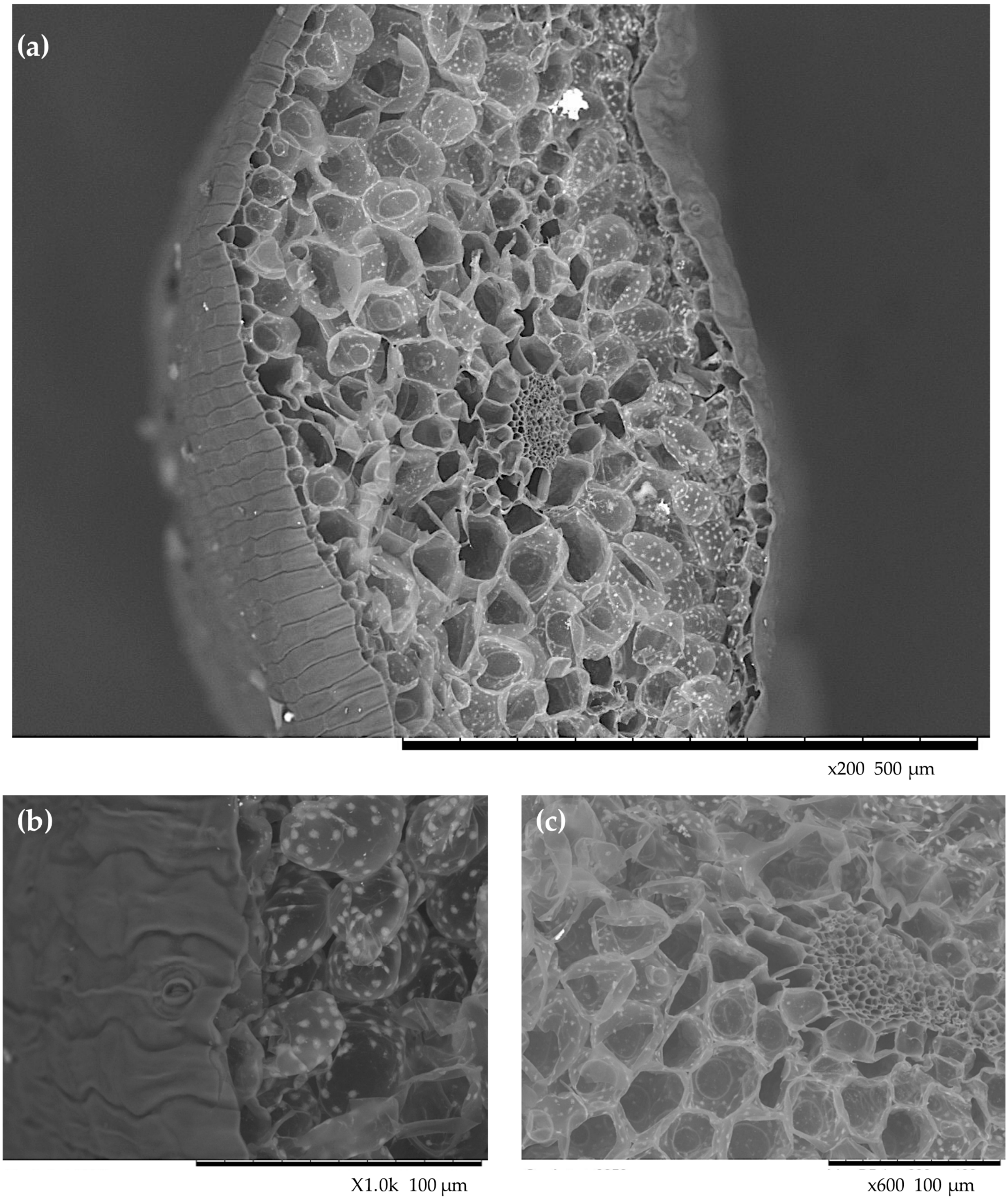

Figure S3. Scanning electron micrograph showing a transverse section of C. quitensis leaf (**a**). A sizeable stomata is visible in (**b**) and vascular bundle in (**c**).
